# Smoking-related gestures as a behavioral dimension of tobacco use: associations with psychological distress and smoking patterns in treatment-seeking smokers

**DOI:** 10.3389/fphar.2026.1904931

**Published:** 2026-07-27

**Authors:** Lorenzo Zamboni, Rebecca Casari, Laura Santin, Alessio Congiu, Anna Guerra, Sara Cappelletti, Silvia Melchiori, Angela Federico, Simone Campagnari, Silvia Carli, Maurizio Valentino Infante, Fabio Lugoboni

**Affiliations:** 1 Department of Internal Medicine, Unit of Addiction Medicine, Azienda Ospedaliera Universitaria Integrata Verona, Verona, Italy; 2 Department of Anaesthesia, Intensive Care and Pain Therapy, University of Verona, Verona, Italy; 3 Department of Neurology, Pederzoli Hospital, Peschiera del Garda (VR), Italy; 4 Department of Internal Medicine, Azienda Ospedaliera Universitaria Integrata Verona, Verona, Italy; 5 Thoracic Surgery Department, University and Hospital Trust-Azienda Ospedaliera Universitaria Integrata, Verona, Italy

**Keywords:** anxiety, depression, nicotine dependence, psychological distress, smoking behavior, smoking gestures

## Abstract

Smoking behavior is influenced not only by nicotine dependence but also by behavioral rituals and psychological factors. The motor routines associated with smoking, such as holding and manipulating the cigarette, may represent an important behavioral component of tobacco addiction. However, the relationship between smoking-related gestures, psychological distress, and smoking behavior has received limited empirical attention. This cross-sectional study analyzed data from 787 treatment-seeking smokers undergoing clinical evaluation for tobacco dependence. Smoking behavior was assessed through the number of cigarettes smoked per day and the Fagerström Test for Nicotine Dependence (FTND). Psychological distress was evaluated using the Zung Self-Rating Anxiety Scale (SAS) and the Zung Self-Rating Depression Scale (SDS). Smoking-related gesturality was measured through a clinical item assessing the perceived importance of smoking gestures. Associations were examined using Spearman correlations, non-parametric group comparisons, and multivariate logistic regression. Gesturality showed a weak but statistically significant association with anxiety (ρ = −0.12, p = 0.001) and depressive symptoms (ρ = −0.13, p < 0.001). Higher gestural involvement was also associated with greater cigarette consumption (ρ = 0.10, p = 0.01). In multivariate analysis, the number of cigarettes smoked per day emerged as the only independent predictor of high gesturality (OR = 1.03, p = 0.02), while anxiety and depressive symptoms showed non-significant trends. Smoking-related gestures appear to reflect a behavioral dimension of tobacco use that is primarily associated with smoking intensity and habitual motor routines, while showing only weak inverse associations with anxiety and depressive symptoms.

## Introduction

Tobacco use remains one of the leading preventable causes of morbidity and mortality worldwide (GBD, 2019 Tobacco Collaborators, 2021). Despite substantial reductions in smoking prevalence in many high-income countries, tobacco consumption continues to represent a major public health challenge, contributing to cardiovascular disease, respiratory disorders, and multiple forms of cancer (Benowitz, 2010; World Health Organization, 2021). Tobacco addiction has traditionally been conceptualized primarily as a pharmacological dependence driven by nicotine exposure. Nicotine acts on nicotinic acetylcholine receptors in the central nervous system, stimulating dopaminergic pathways involved in reward and reinforcement, thereby contributing to the development and maintenance of dependence (Benowitz, 2010; Dani and De Biasi, 2001). However, increasing evidence suggests that tobacco use cannot be fully understood solely through the pharmacological effects of nicotine.

In addition to its biochemical mechanisms, smoking behavior involves a complex set of learned routines, environmental cues, and sensorimotor experiences that may reinforce tobacco use independently of nicotine intake (Carter and Tiffany, 1999). The repetitive motor patterns associated with smoking—such as holding the cigarette, bringing it to the mouth, inhaling smoke, and manipulating the cigarette between the fingers—constitute ritualized behaviors that become strongly embedded within daily routines (Rose, 2006). These ritualistic components of smoking may provide sensory and behavioral reinforcement that contributes to the persistence of tobacco use even when nicotine exposure is reduced. Indeed, behavioral models of addiction emphasize that repeated actions can gradually evolve into habits through reinforcement processes involving neural circuits linking the basal ganglia and prefrontal cortex (Everitt and Robbins, 2016; Robbins and Costa, 2017). Within this perspective, smoking can be understood not only as a pharmacological dependence but also as a habitual behavioral pattern sustained by environmental and emotional triggers.

The importance of behavioral and ritualistic aspects of smoking is also supported by research examining alternative nicotine delivery systems. Several studies have shown that smokers often value the sensorimotor aspects of smoking, including the hand-to-mouth movement and the inhalation ritual, which may contribute to the subjective satisfaction derived from smoking (Rose, 2006; Shiffman et al., 2014). This phenomenon has also been observed in studies investigating electronic cigarettes and other smoking substitutes, where the replication of smoking gestures has been identified as an important factor influencing user acceptance and perceived effectiveness as cessation aids (Etter and Bullen, 2011; Caponnetto et al., 2013). These findings suggest that the motor and ritualistic dimensions of smoking may play a meaningful role in reinforcing tobacco use behavior.

Beyond behavioral reinforcement, psychological factors also contribute significantly to tobacco use. A large body of literature has documented strong associations between smoking and psychiatric symptoms, particularly anxiety and depressive disorders (Fluharty et al., 2017; Taylor et al., 2014).

**FIGURE 1:**
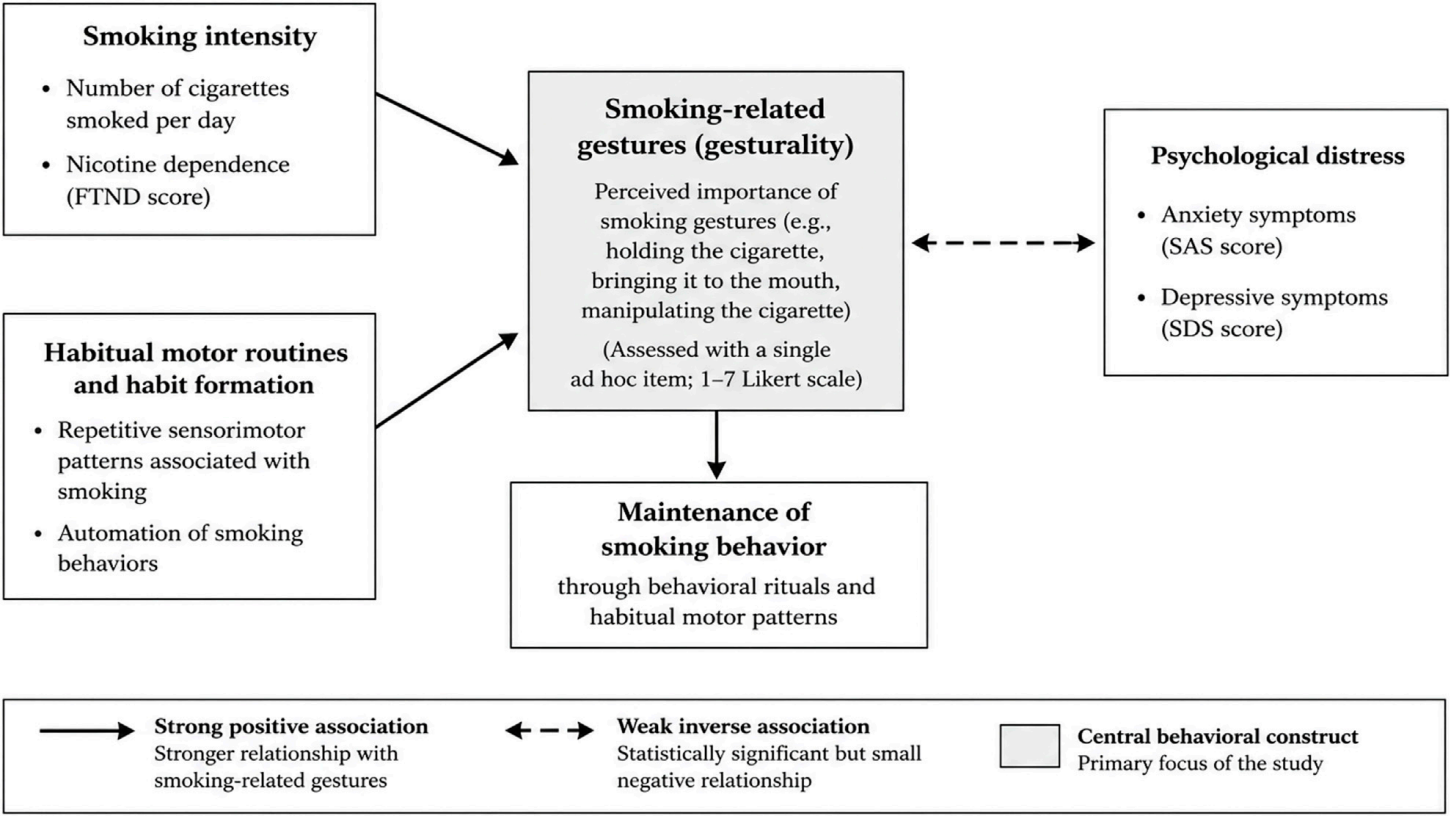
Conceptual framework of smoking-related gestures in tobacco use.

The strong correlation observed between anxiety and depressive symptoms in this sample is consistent with previous research highlighting the frequent co-occurrence of these dimensions within broader constructs of psychological distress (Fluharty et al., 2017). Figure 1 illustrates the conceptual framework underlying the present study and the hypothesized relationships among smoking-related gestures, smoking intensity, habitual motor routines, psychological distress, and maintenance of smoking behavior.

Smokers frequently report using cigarettes as a means of coping with emotional distress or regulating negative affective states (Kassel et al., 2003; Morissette et al., 2007). Laboratory studies have shown that nicotine administration can transiently alleviate subjective stress and anxiety, reinforcing the perception that smoking provides emotional relief (Parrott, 1999). However, this relationship is complex and bidirectional. While smokers often perceive cigarettes as helpful in managing stress, longitudinal research suggests that smoking may actually exacerbate anxiety and depressive symptoms over time, partly due to withdrawal cycles and neurobiological changes associated with chronic nicotine exposure (Taylor et al., 2014).

Although anxiety and depression have been extensively investigated in relation to smoking behavior, recent evidence suggests that additional psychological dimensions may contribute to smoking maintenance and difficulties in cessation. In particular, boredom proneness has been associated with greater difficulty quitting smoking, while affective temperaments, especially cyclothymic temperament, may increase vulnerability to persistent smoking through their relationship with emotional dysregulation and maladaptive coping strategies. These findings support a multidimensional conceptualization of tobacco dependence, suggesting that emotional distress, personality-related characteristics, and behavioral rituals may jointly contribute to the maintenance of smoking behavior and should therefore be considered when investigating the behavioral components of tobacco addiction (Iannuzzo et al., 2025).

The interaction between psychological distress and smoking behavior may also involve the behavioral rituals associated with smoking. From a behavioral perspective, smoking gestures may function as conditioned responses that become linked to emotional states. For instance, individuals experiencing anxiety or tension may engage in the motor routines of smoking as a learned behavioral strategy to modulate emotional discomfort. Over time, these gestures may become part of a habitual loop in which emotional triggers activate the motor sequence associated with smoking, thereby reinforcing the behavior independently of nicotine intake. This interpretation is consistent with contemporary neurobehavioral models of addiction, which highlight the role of conditioned cues and habitual action patterns in maintaining substance use behaviors (Everitt and Robbins, 2016).

Although the role of psychological distress in smoking behavior has been widely investigated, relatively few studies have specifically examined the relationship between emotional factors and the gestural or ritualistic components of smoking. Understanding this relationship may provide valuable insights into the behavioral mechanisms that sustain tobacco use. Smoking gestures may represent an observable behavioral manifestation of the interaction between emotional states and habitual motor patterns. As such, they may help explain why some smokers continue to experience strong urges to smoke even when pharmacological cravings are relatively mild.

Recent research has begun to explore this dimension of smoking behavior. In a clinical sample of treatment-seeking smokers, Zamboni et al. (2025) reported a significant association between anxiety symptoms and the perceived importance of smoking gestures, suggesting that these behavioral routines may be linked to emotional regulation processes. These preliminary findings highlighted the potential relevance of smoking-related gestures as a behavioral component of tobacco use, although the study was conducted in a relatively small sample and further research is needed to clarify the relationship between gestural behavior, psychological distress, and smoking patterns.

In addition, most studies examining smoking behavior have focused primarily on nicotine dependence and cigarette consumption, while less attention has been paid to the behavioral rituals associated with smoking. Yet these rituals may represent an important dimension of tobacco use that is not captured by traditional measures of nicotine dependence. For example, instruments such as the Fagerström Test for Nicotine Dependence (FTND) primarily assess physiological and behavioral indicators of dependence, including time to first cigarette and daily cigarette consumption (Heatherton et al., 1991). While these measures are essential for assessing nicotine addiction, they may not fully reflect the motor and ritualistic aspects of smoking behavior that may contribute to its persistence.

A better understanding of smoking-related gestures may therefore have important implications for both research and clinical practice. If gestural patterns are associated with psychological distress or habitual smoking behavior, they could represent a behavioral target for smoking cessation interventions. Behavioral substitution strategies, mindfulness-based approaches, and interventions aimed at disrupting habitual routines may help address the non-pharmacological components of tobacco dependence (Jackson et al., 2021).

While nicotine dependence remains a central component of tobacco addiction, the ritualistic and motor aspects of smoking may represent an additional dimension influencing smoking behavior and its relationship with psychological distress. Behavioral models of addiction emphasize that repeated motor routines and conditioned cues may contribute to the maintenance of substance use beyond purely pharmacological reinforcement (Everitt and Robbins, 2016; Rose, 2006; Volkow et al., 2016). The present study aimed to further investigate the relationship between smoking-related gesturality, psychological distress, and smoking behavior in a large clinical sample of treatment-seeking smokers. In particular, we examined whether gestural involvement was associated with symptoms of anxiety and depression, as well as with smoking intensity and nicotine dependence severity. By exploring these relationships in a large dataset, this study seeks to contribute to a better understanding of the behavioral and psychological dimensions underlying tobacco use.

## Methods

### Study design and participants

This study was based on a cross-sectional analysis of clinical data collected from smokers seeking treatment for tobacco dependence at a specialized addiction service.

Participants were recruited from a specialized outpatient addiction service within a university-affiliated hospital setting. The service provides multidisciplinary treatment for tobacco use disorder, including medical, psychological, and behavioral interventions.

All participants were treatment-seeking smokers who voluntarily accessed the service for smoking cessation. As such, the sample represents a clinical population characterized by moderate to high levels of nicotine dependence and motivation to quit smoking.

Clinical and demographic data were collected as part of routine clinical practice and subsequently anonymized for research purposes. This real-world clinical dataset reflects the characteristics of smokers actively seeking treatment, although it may not be fully representative of the general smoking population.

All participants provided informed consent for the use of anonymized clinical data for research purposes in accordance with institutional procedures and ethical guidelines for observational research.

### Ethics statement

This study was conducted in accordance with the principles of the Declaration of Helsinki and received approval from the local Ethics Committee for Clinical Trials of the Provinces of Verona and Rovigo. The research protocol was reviewed and approved under protocol number 683CESC.

All participants were informed about the objectives and procedures of the study and provided written informed consent prior to participation.

### Measures

#### Sociodemographic and smoking-related variables

Basic demographic information was collected during the clinical assessment, including age, sex, and employment status.

Smoking behavior was assessed using the following variables.

- Age at smoking initiation, defined as the age at which the individual began regular cigarette smoking.
- Number of cigarettes smoked per day, representing the average daily cigarette consumption at the time of the assessment.

These variables were used as indicators of smoking intensity and smoking history.

#### Nicotine dependence

Nicotine dependence was evaluated using the Fagerström Test for Nicotine Dependence (FTND) (Heatherton et al., 1991). The FTND is a widely used six-item questionnaire designed to assess the severity of nicotine dependence.

Scores range from 0 to 10, with higher scores indicating greater nicotine dependence. In accordance with standard cut-offs, dependence levels were categorized as follows.

- 0–2: very low dependence
- 3–4: low dependence
- 5: moderate dependence
- 6–7: high dependence
- 8–10: very high dependence

In the present study, FTND scores were analyzed both as a continuous variable and as categorical levels of dependence for descriptive purposes.

#### Psychological distress

Symptoms of anxiety and depression were assessed using the Zung Self-Rating Anxiety Scale (SAS) and the Zung Self-Rating Depression Scale (SDS) (Zung, 1971; Dunstan et al., 2020).

The SAS is a 20-item self-report instrument designed to evaluate the presence and severity of anxiety symptoms. Total scores range from 20 to 80, with higher scores indicating greater levels of anxiety. Based on established thresholds, anxiety severity can be interpreted as.

- 20–44: normal range
- 45–59: mild to moderate anxiety
- 60–74: marked anxiety
- ≥75: severe anxiety

The SDS is a parallel 20-item self-report scale assessing depressive symptoms. Total scores range from 20 to 80, with higher scores reflecting greater depressive symptomatology. Standard interpretative thresholds include.

- 20–49: normal range
- 50–59: mild depression
- 60–69: moderate depression
- ≥70: severe depression

In the present study, both SAS and SDS scores were analyzed primarily as continuous variables, while categorical thresholds were used for descriptive analyses.

These instruments were selected due to their widespread use in clinical settings and their suitability for rapid assessment of psychological distress in routine practice.

### Smoking-related gesturality

Smoking-related gesturality was assessed using a single clinical item specifically developed for the present study, as no validated instrument assessing this construct was available at the time of study design. Participants were asked: “How important is the gestural aspect of smoking for you when you smoke (e.g., holding the cigarette between the fingers, bringing it to the mouth, manipulating it while smoking)?” Responses were recorded on a seven-point Likert scale ranging from 1 (“not important at all”) to 7 (“very important”), with higher scores indicating greater perceived importance of smoking-related gestures. The English version of the item is provided as Supplementary Material 1. Although this measure has not undergone formal psychometric validation, it was developed to provide a pragmatic clinical assessment of the subjective relevance of smoking-related motor and ritualistic behaviors in treatment-seeking smokers.

### Statistical analysis

Descriptive statistics were used to summarize demographic characteristics, smoking behavior, and psychological measures. Continuous variables are presented as means and standard deviations, while categorical variables are reported as frequencies and percentages.

Given the ordinal nature of some variables and the potential non-normal distribution of gesturality scores, non-parametric statistical tests were used for several analyses.

Associations between gesturality and continuous variables—including anxiety (SAS), depression (SDS), cigarettes smoked per day, FTND score, age, and age at smoking initiation—were evaluated using Spearman rank correlation coefficients.

Differences in gesturality across anxiety severity categories and smoking intensity groups were examined using the Kruskal–Wallis test.

To further explore the characteristics associated with smoking-related gestures, participants were divided into low versus high gesturality groups based on the median gesturality score. Comparisons between these groups were conducted using Mann–Whitney U tests for continuous variables.

Finally, a multivariate logistic regression model was performed to identify independent predictors of high gesturality. The model included the following predictors.

- anxiety symptoms (SAS score)
- depressive symptoms (SDS score)
- nicotine dependence (FTND score)
- number of cigarettes smoked per day
- age
- sex

Results of the regression analysis are reported as odds ratios (ORs) with 95% confidence intervals (CIs).

All statistical analyses were conducted using standard statistical software, and statistical significance was defined as p < 0.05.

Because anxiety (SAS) and depression (SDS) scores were expected to be strongly correlated, multicollinearity diagnostics were performed before fitting the multivariate logistic regression model. Variance inflation factors (VIF) and tolerance values were calculated for all predictors included in the model.

## Results

The final dataset comprised 787 participants who were evaluated for smoking behavior and associated psychological variables. Due to missing data in some variables, the number of observations included in specific analyses ranged between 688 and 690 participants.

Participants had a mean age of approximately 51.8 years. The average age at smoking initiation was around 17–18 years. The mean number of cigarettes smoked per day was approximately 18–20 cigarettes.

Nicotine dependence, assessed using the Fagerström Test for Nicotine Dependence (FTND), showed a mean score of 5.9, indicating moderate to high nicotine dependence on average.

Regarding psychological variables, the mean Self-Rating Anxiety Scale (SAS) score was 26.5, while the mean Self-Rating Depression Scale (SDS) score was 27.3, suggesting that, on average, participants fell within the normal range for both anxiety and depression symptoms.

The demographic, clinical, and smoking-related characteristics of the sample are reported in Table 1.

**TABLE 1:** Sample characteristics.

Variable	Mean ± SD / N (%)
Participants	787
Age (years)	51.8 ± 12.6
Age at smoking onset (years)	17.4 ± 3.9
Cigarettes per day	18.9 ± 8.2
FTND score	5.9 ± 2.1
SAS score	26.5 ± 8.3
SDS score	27.3 ± 9.1
Gesturality score	4.1 ± 1.6
FTND categories	
Category	N (%)
Very low (0–2)	74 (9.4%)
Low (3–4)	121 (15.4%)
Moderate (5)	156 (19.8%)
High (6–7)	275 (35.0%)
Very high (8–10)	161 (20.4%)

### Correlations between gesturality and clinical variables

Spearman rank correlation analyses were conducted to examine the relationships between smoking-related gesturality and psychological as well as behavioral variables.

A weak but statistically significant negative correlation emerged between gesturality and anxiety symptoms (SAS) (ρ = −0.12, *p* = 0.001). Similarly, a small negative correlation was observed between gesturality and depressive symptoms (SDS) (ρ = −0.13, *p* < 0.001).

Conversely, a positive correlation was found between gesturality and the number of cigarettes smoked per day (ρ = 0.10, *p* = 0.01), indicating that individuals who smoked more cigarettes reported slightly higher levels of smoking-related gestural involvement.

No significant association was found between gesturality and nicotine dependence severity as measured by the FTND score (ρ = 0.04, *p* = 0.30). Likewise, gesturality was not significantly correlated with age (ρ = −0.04, *p* = 0.26) or age at smoking onset (ρ = −0.04, *p* = 0.24).

A very strong correlation was observed between anxiety and depressive symptoms (SAS and SDS) (ρ = 0.905, *p* < 0.001), indicating substantial overlap between the two psychological constructs within this sample.

The results of the Spearman correlation analyses are summarized in Table 2.

**TABLE 2:** Spearman correlations between gesturality and clinical variables.

Variable	Spearman ρ	p-value
SAS (anxiety)	−0.12	0.001
SDS (depression)	−0.13	<0.001
Cigarettes per day	0.10	0.01
FTND score	0.04	0.30
Age	−0.04	0.26
Age at smoking onset	−0.04	0.24
Correlation between psychological scales		
SAS – SDS	0.905	<0.001

### Comparison across anxiety severity categories

Participants were categorized according to clinically established SAS thresholds (normal anxiety, mild–moderate anxiety, elevated anxiety, and extremely elevated anxiety). A Kruskal–Wallis test was performed to assess differences in gesturality across these groups.

The analysis revealed a statistically significant difference in gesturality across anxiety severity levels (χ^2^ = 9.43, *p* = 0.024), indicating that the degree of smoking-related gestural involvement varied across anxiety categories.

### Gesturality and smoking intensity

To further explore the relationship between gesturality and smoking behavior, participants were categorized according to the number of cigarettes smoked per day.

- ≤10 cigarettes/day
- 11–20 cigarettes/day
- >20 cigarettes/day

A Kruskal–Wallis test revealed a significant difference in gesturality across smoking intensity groups (*p* = 0.02), with higher gesturality scores observed among heavy smokers.

No significant differences in gesturality were observed across FTND dependence categories (Kruskal–Wallis test, p > 0.05), suggesting that smoking-related gestures may not be directly related to the severity of nicotine dependence.

Interestingly, nicotine dependence severity measured by the FTND did not emerge as a significant predictor of gesturality.

### Analysis using dichotomized gesturality

Gesturality was further dichotomized using the median value to create two groups: low gesturality and high gesturality.

Comparisons between these groups showed that participants with higher gesturality smoked significantly more cigarettes per day (approximately 20 vs. 17 cigarettes/day, *p* = 0.01).

Small but statistically significant differences were also observed for psychological variables. Individuals with higher gesturality displayed slightly lower mean anxiety scores (SAS = 26 vs. 27, *p* = 0.04) and lower depressive symptoms (SDS = 26 vs. 28, *p* = 0.03).

Comparisons between participants with low and high gesturality are reported in Table 3.

**TABLE 3:** Comparison between participants with low and high gesturality.

Variable	Low gesturality	High gesturality	p-value
Cigarettes/day	17.1 ± 7.9	20.2 ± 8.3	0.01
SAS	27.1 ± 8.5	26.0 ± 8.1	0.04
SDS	28.1 ± 9.3	26.3 ± 8.8	0.03
FTND	5.8 ± 2.2	6.0 ± 2.1	0.44
Age	52.1 ± 12.3	51.4 ± 12.8	0.51

### Multivariate analysis

A multivariate logistic regression model was conducted to identify independent predictors of high gesturality. The model included the following variables.

- SAS score
- SDS score
- FTND score
- number of cigarettes per day
- age
- sex

Prior to the regression analysis, multicollinearity diagnostics were performed. As expected, anxiety (SAS) and depression (SDS) showed moderate multicollinearity (VIF = 6.48 and 6.43; tolerance = 0.154 and 0.156, respectively), reflecting the substantial overlap between the two psychological measures. All remaining predictors showed VIF values close to 1.0 and tolerance values above 0.96, indicating no relevant multicollinearity among these variables. Since VIF values remained below the commonly accepted threshold of 10, all variables were retained in the final regression model.

In the multivariate model, the number of cigarettes smoked per day emerged as the only independent predictor of high gesturality (OR = 1.03, *p* = 0.02).

Anxiety and depressive symptoms showed a trend toward association but did not reach statistical significance (SAS: *p* = 0.09; SDS: *p* = 0.07). Nicotine dependence severity measured by the FTND was not significantly associated with gesturality (*p* = 0.44).

The results of the multivariate logistic regression model are presented in Table 4.

**TABLE 4:** Multivariate logistic regression predicting high gesturality.

Predictor	OR	95% CI	p-value
Cigarettes/day	1.03	1.01–1.06	0.02
SAS	0.98	0.95–1.01	0.09
SDS	0.97	0.94–1.01	0.07
FTND	1.02	0.96–1.08	0.44
Age	0.99	0.98–1.01	0.31
Sex	1.05	0.79–1.39	0.74

### Summary of findings

Overall, the analyses indicate that smoking-related gesturality is weakly associated with psychological distress but is more consistently related to smoking intensity, as reflected by the number of cigarettes smoked per day. No significant independent relationship was observed between gesturality and nicotine dependence severity.

## Discussion

The present study explored the relationship between smoking-related gesturality, psychological distress, and smoking behavior in a large clinical sample of treatment-seeking smokers. Overall, the findings suggest that smoking-related gestures primarily represent a behavioral dimension of tobacco use, showing stronger associations with smoking intensity than with current psychological distress. Although weak inverse associations with anxiety and depressive symptoms were observed in the bivariate analyses, smoking intensity emerged as the only independent predictor of gestural involvement in the multivariate model. Conversely, nicotine dependence severity, as measured by the Fagerström Test for Nicotine Dependence (FTND), was not independently associated with gesturality. These findings support the view that smoking gestures represent a distinct behavioral dimension of tobacco use that cannot be fully explained by pharmacological dependence alone.

The role of behavioral and sensorimotor components in tobacco addiction has been increasingly recognized in the literature. Smoking is not merely a pharmacological behavior driven by nicotine intake but also involves a complex sequence of motor routines, environmental cues, and conditioned responses (Benowitz, 2010). The repetitive act of bringing a cigarette to the mouth, handling the cigarette, and observing the smoke may provide sensory and proprioceptive feedback that reinforces the behavior independently of nicotine delivery. This perspective aligns with contemporary neurobehavioral models of addiction, which conceptualize substance use as the result of both pharmacological reinforcement and learned behavioral patterns (Everitt and Robbins, 2016).

A growing body of research has begun to specifically examine the role of smoking-related gestures in tobacco use. Previous work has suggested that the ritualized motor aspects of smoking—such as holding the cigarette, bringing it to the mouth, and manipulating it during consumption—may represent meaningful components of the smoking experience beyond nicotine delivery. In a clinical sample of treatment-seeking smokers, Zamboni et al. (2025) reported a significant association between anxiety symptoms and the perceived importance of smoking gestures, suggesting that these behaviors may function as a form of behavioral self-regulation in individuals experiencing psychological distress. The present findings suggest that psychological factors may contribute to the subjective experience of smoking-related gestures; however, this contribution appears to be modest and secondary to smoking intensity and habitual motor routines. Together, these findings support the view that smoking-related gesturality reflects a multidimensional behavioral construct rather than a direct manifestation of psychological distress alone.

Within this framework, smoking gestures can be interpreted as part of a habitual behavioral loop. According to habit-based models of addiction, repeated behaviors become automated through cue–routine–reward cycles involving the basal ganglia and related neural circuits (Everitt and Robbins, 2016). In the context of tobacco use, environmental and emotional cues may activate habitual smoking routines, while the motor sequence of smoking provides a behavioral response reinforced by both pharmacological and non-pharmacological rewards. The calming or regulatory effects associated with the ritualized act of smoking may therefore contribute to the persistence of tobacco use even in individuals with relatively low levels of nicotine dependence.

Although our initial hypothesis proposed that greater psychological distress would be associated with stronger smoking-related gesturality, the present findings did not support this assumption. Instead, the observed inverse association suggests that smoking-related gestures may constitute a behavioral dimension that is at least partially independent from current anxiety and depressive symptom severity.

These findings suggest that emotional distress alone is unlikely to explain individual differences in smoking-related gesturality. Rather, gesturality appears to reflect a behavioral dimension that may emerge from the interaction between habitual motor routines, smoking intensity, and, to a lesser extent, psychological factors.

Previous studies have highlighted the strong link between tobacco use and psychiatric symptoms, particularly anxiety and mood disorders (Taylor et al., 2014). Smokers frequently report using cigarettes as a means of emotional regulation, and laboratory studies have shown that nicotine administration can transiently reduce subjective stress and anxiety levels (Parrott, 1999). However, it has also been suggested that part of the perceived anxiolytic effect of smoking may be attributable to the ritualistic aspects of the behavior rather than nicotine itself.

Indeed, the sensorimotor experience of smoking has been described as a potential source of reinforcement independent of pharmacological effects. For example, research on alternative nicotine delivery systems has suggested that the replication of smoking gestures—such as the hand-to-mouth movement—plays an important role in user satisfaction and adherence to smoking substitutes (Perkins, 2001; Etter and Bullen, 2011). Similarly, studies examining electronic cigarettes have shown that behavioral similarity to conventional smoking may contribute significantly to their perceived effectiveness as cessation aids (Caponnetto et al., 2013). These observations support the hypothesis that the gestural and ritualistic components of smoking may function as behavioral reinforcers in their own right.

Another notable finding of the present study is the association between gesturality and smoking intensity, as reflected by the number of cigarettes smoked per day. Individuals who reported higher levels of gestural involvement also tended to smoke more cigarettes daily. This relationship suggests that gesturality may be partly shaped by the frequency of smoking behavior itself. Repeated engagement in the motor routines associated with smoking may strengthen these patterns over time through mechanisms of behavioral reinforcement and habit formation. In this sense, gesturality may represent both a consequence and a contributor to smoking persistence.

Interestingly, nicotine dependence severity measured by the FTND did not emerge as a significant predictor of gesturality.

The absence of a clear association between gesturality and FTND scores further supports the hypothesis that smoking-related gestures may reflect behavioral and ritualistic aspects of smoking rather than the pharmacological dimension of nicotine dependence.

This finding reinforces the notion that the behavioral dimension of smoking may be partially independent from biochemical dependence. The FTND primarily captures aspects of physical dependence, such as time to first cigarette and difficulty refraining from smoking in restricted environments (Heatherton et al., 1991). While these factors are important indicators of nicotine addiction, they may not adequately capture the behavioral and ritualistic components of smoking behavior.

Taken together, these results support a multidimensional view of tobacco addiction. Contemporary models increasingly emphasize the interplay between pharmacological, psychological, and behavioral factors in maintaining substance use (Koob and Le Moal, 2008). In this context, smoking-related gestures may represent a behavioral phenotype reflecting both emotional regulation processes and the repetitive motor patterns associated with habitual smoking.

From a clinical perspective, these findings have potential implications for smoking cessation interventions. Traditional pharmacological approaches primarily target nicotine dependence and withdrawal symptoms. However, smokers who derive reinforcement from the behavioral rituals of smoking may experience persistent urges even when pharmacological cravings are adequately managed. Behavioral interventions that address the ritualistic aspects of smoking—such as behavioral substitution strategies, mindfulness-based techniques, or the use of smoking substitutes—may therefore represent valuable adjuncts to pharmacotherapy. In particular, devices that replicate the motor patterns of smoking without delivering nicotine may help reduce the behavioral component of dependence during the cessation process.

More specifically, smokers reporting high levels of gestural involvement may benefit from interventions specifically targeting habitual motor routines, including behavioral substitution strategies, cue-exposure techniques, mindfulness-based interventions, and habit-reversal approaches. Assessing the subjective importance of smoking-related gestures during clinical evaluation may help identify patients for whom addressing the behavioral component of smoking could enhance the effectiveness of conventional pharmacological treatments. These interventions may be particularly useful in individuals whose smoking behavior is maintained not only by nicotine dependence but also by highly automatized sensorimotor routines.

The present study has several limitations that should be considered when interpreting the results. First, the cross-sectional design prevents causal conclusions regarding the relationship between anxiety, smoking behavior, and gesturality.

It remains unclear whether anxiety promotes greater reliance on smoking-related gestures, or whether habitual engagement in smoking rituals influences emotional regulation processes. Longitudinal studies are needed to clarify the temporal dynamics of these relationships.

Therefore, gesturality as assessed in the present study should be interpreted as a subjective proxy of the behavioral and ritualistic components of smoking rather than a direct or objective measure of motor behavior. This limitation may have attenuated the strength of the observed associations and could partly explain the modest effect sizes identified.

Future studies employing objective behavioral assessments, ecological momentary assessment, or validated multi-item scales will be essential to better characterize the role of smoking-related gestures in tobacco use and to clarify their relationship with psychological distress and smoking patterns.

Another limitation concerns the assessment of smoking-related gesturality. In the present study, gestural involvement was evaluated using a single clinical item rather than a standardized multi-item scale. Although this approach allowed for the rapid assessment of the perceived importance of smoking gestures in a clinical setting, future studies would benefit from the development and validation of dedicated instruments designed to measure smoking-related motor rituals more comprehensively.

Despite these limitations, the present study contributes to the growing body of literature highlighting the behavioral dimensions of tobacco use. By examining smoking-related gestures in a large clinical sample, this research provides further evidence that smoking behavior cannot be fully understood solely through the lens of nicotine dependence. Instead, the ritualistic and sensorimotor aspects of smoking appear to play a meaningful role in the maintenance of tobacco use.

In conclusion, smoking-related gestures appear to represent a behavioral dimension of tobacco use influenced by both emotional factors and smoking patterns. Anxiety symptoms showed a modest but consistent association with gestural involvement, suggesting that emotional factors should be considered when examining the behavioral rituals of smoking. At the same time, smoking intensity also appeared to contribute to the expression of these behaviors, indicating that gesturality may emerge from the interaction between emotional regulation processes and the repetitive motor patterns inherent in smoking behavior.

The model illustrates the proposed relationships between psychological distress (anxiety and depression), smoking-related gestures, and smoking behavior. Smoking-related gestures may represent a behavioral component of tobacco use influenced by emotional factors and reinforced by the habitual repetition of smoking behavior. Nicotine dependence severity, as measured by the FTND, did not show a significant association with gesturality in the present study.

## Conclusion

Smoking-related gestures appear to represent a behavioral dimension of tobacco use that is primarily associated with smoking intensity and habitual motor routines, while showing only weak inverse associations with anxiety and depressive symptoms. In this large clinical sample of treatment-seeking smokers, smoking intensity emerged as the only independent predictor of high gestural involvement, whereas psychological distress was not independently associated with gesturality after adjustment for other clinical variables. These findings support the view that smoking behavior is maintained not only by pharmacological dependence but also by behavioral rituals and learned motor patterns. A better understanding of these behavioral components may contribute to a more comprehensive conceptualization of tobacco addiction and may help inform smoking cessation interventions targeting both the pharmacological and behavioral dimensions of smoking.

## Data Availability

The raw data supporting the conclusions of this article will be made available by the authors, without undue reservation.
