# Supplementary material for: Smoking-related gestures as a behavioral dimension of tobacco use: associations with psychological distress and smoking patterns in treatment-seeking smokers
Source: Front Pharmacol. 2026 Jul 27;17:1904931. doi: 10.3389/fphar.2026.1904931 (PMC13453294; doi:10.3389/fphar.2026.1904931)
Supplement: Supplementary file 1 [file Supplementaryfile1.docx]

Supplementary material

Gesture question:

“How important is the gestural aspect of smoking for you when you smoke (e.g., holding the cigarette between the fingers, bringing it to the mouth, manipulating it while smoking)?”

Response scale:

1 = Not important at all
2 = Slightly important
3 = Mildly important
4 = Moderately important
5 = Quite important
6 = Very important
7 = Extremely important
